# Supplementary material for: BugSplit enables genome-resolved metagenomics through highly accurate taxonomic binning of metagenomic assemblies
Source: Commun Biol. 2022 Feb 22;5:151. doi: 10.1038/s42003-022-03114-4 (PMC8864044; doi:10.1038/s42003-022-03114-4)
Supplement: Supplementary file 1 — Supplemental Material [file 42003_2022_3114_MOESM1_ESM.pdf]

Supplementary Material

BugSplit enables genome-resolved metagenomics through highly accurate taxonomic binning of metagenomic assemblies

Induja Chandrakumar[1], Nick P.G. Gauthier[2], Cassidy Nelson[3], Michael B. Bonsall[3], Kerstin Locher[4,5], Marthe Charles[4,5], Clayton MacDonald[4,5], Mel Krajden[5,6], Amee R. Manges[6,7], Samuel D. Chorlton[1,5]

[1]BugSeq Bioinformatics Inc, Vancouver, British Columbia, Canada

[2]Department of Microbiology and Immunology, University of British Columbia, Vancouver, British Columbia, Canada

[3]Mathematical Ecology Research Group, Department of Zoology, University of Oxford, Oxford, United Kingdom

[4]Division of Medical Microbiology, Vancouver General Hospital, Vancouver, British Columbia, Canada

[5]Department of Pathology and Laboratory Medicine, University of British Columbia, Vancouver, British Columbia, Canada

[6]British Columbia Centre for Disease Control, Vancouver, British Columbia, Canada

[7]School of Population and Public Health, University of British Columbia, Vancouver, British Columbia, Canada

Table of Contents

Supplementary Tables 3
Supplementary Figures 7
Supplementary Note 1 10

Supplementary Tables

Quality of bins: all bins have the same weight				
Tool	Gold standard	BugSplit	MMseqs2	DIAMOND+ MEGAN-LR
Average purity (bp)	1	0.801	0.754	0.749
Average purity (seq)	1	0.8	0.754	0.749
Average completeness (bp)	1	0.593	0.149	0.268
Average completeness (seq)	1	0.561	0.18	0.244
F1 score (bp)	1	0.655	0.229	0.393
F1 score (seq)	1	0.636	0.269	0.364
Std error of average purity (bp)	0	0.057	0.002	0.012
Std error of average purity (seq)	0	0.057	0.002	0.012
Std error of average completeness (bp)	0	0.076	0.067	0.105
Std error of average completeness (seq)	0	0.088	0.064	0.094
Quality for sample				
Accuracy (bp)	1	0.681	0.192	0.38
Accuracy (seq)	1	0.637	0.265	0.351
Misclassification rate (bp)	0	0.066	0.219	0.252
Misclassification rate (seq)	0	0.099	0.235	0.263
Purity (bp)	1	0.934	0.781	0.748
Purity (seq)	1	0.901	0.765	0.737
Completeness (bp)	1	0.681	0.192	0.38
Completeness (seq)	1	0.637	0.265	0.351
F1 score for sample (bp)	1	0.73	0.282	0.496
F1 score for sample (seq)	1	0.701	0.355	0.461
Rand index (bp)	1	0.987	0.996	0.98
Rand index (seq)	1	0.962	0.991	0.954
Adjusted Rand index (bp)	1	0.965	0.938	0.881
Adjusted Rand index (seq)	1	0.918	0.928	0.874
Percentage of binned bp	1	0.7	0.239	0.457
Percentage of binned sequences	1	0.669	0.332	0.426
UniFrac (bp)	0	0.75	1.431	1.934
UniFrac (seq)	0	0.813	1.429	1.7

Supplementary Table 1: AMBER output for species-level binning performance. AMBER was run comparing BugSplit, MMSeqs2 and DIAMOND+MEGAN-LR across four metagenomic datasets.

	**Zymo Even (ONT)**	**Zymo Log (ONT)**	**Zymo Gut (PacBio)**	**CAMI High-Complexity (Illumina)**
**BugSplit**	136	79	65	208
**DIAMOND+MEGAN-LR**	1003	687	550	185
**MMseqs2**	226	221	109	14

Supplementary Table 2: Execution time in minutes of BugSplit, DIAMOND+MEGAN-LR and MMseqs2 across four benchmarking datasets. All tools were run on AWS r5a.24xl instances, containing 96 CPUs and 768Gb of RAM.

Presence/absence of taxa						
**Tool**	**Gold standard**	**BugSeq**	**BugSplit**	**Centrifuge**	**DIAMND+ MEGAN-LR**	**MMseqs2**
**Completeness**	1 (0)	0.95 (0.05)	0.749 (0.126)	0.855 (0.0451)	0.443 (0.0431)	0.476 (0.0392)
**Purity**	1 (0)	0.129 (0.0438)	0.894 (0.0187)	0.0032 (0.00155)	0.939 (0.0606)	0.939 (0.0606)
**F1 score**	1 (0)	0.224 (0.0664)	0.806 (0.0749)	0.00636 (0.00306)	0.595 (0.0238)	0.627 (0.0286)
**True positives**	70 (57.7)	9.5 (0.5)	9 (1.53)	10.3 (1.33)	5.67 (1.67)	6 (1.53)
**False positives**	0 (0)	75 (32)	1 (0)	4.31e+03 (1.13e+03)	0.667 (0.667)	0.667 (0.667)
**False negatives**	0 (0)	0.5 (0.5)	3.33 (1.76)	2 (1)	6.67 (0.667)	6.33 (0.882)
**Jaccard index**	1 (0)	0.128 (0.0422)	0.689 (0.112)	0.0032 (0.00154)	0.425 (0.0246)	0.458 (0.0299)
**Abundance estimates**						
**Weighted UniFrac error**	0 (0)	2.04 (0.517)	1.97 (0.762)	2.4 (0.522)	3.35 (0.771)	2.65 (0.431)
**Unweighted UniFrac error**	0 (0)	1.93e+03 (1e+03)	39.7 (5.21)	3.03e+04 (7.77e+03)	62.3 (7.8)	62 (7.81)
**L1 norm error**	0 (0)	0.195 (0.122)	0.367 (0.216)	0.467 (0.243)	0.844 (0.0367)	0.838 (0.0399)
**Bray-Curtis distance**	0 (0)	0.0989 (0.0619)	0.236 (0.157)	0.255 (0.131)	0.716 (0.0673)	0.708 (0.0701)
**Alpha diversity**						
**Taxon counts**	70 (57.7)	84.5 (32.5)	10 (1.53)	4.32e+03 (1.13e+03)	6.33 (2.33)	6.67 (2.19)
**Shannon diversity**	1.64 (0.417)	1.22 (0.877)	1.25 (0.46)	1.58 (0.635)	0.528 (0.179)	0.535 (0.179)
**Shannon equitability**	0.555 (0.179)	0.263 (0.177)	0.532 (0.18)	0.196 (0.0832)	0.295 (0.0645)	0.282 (0.0587)

Supplementary Table 3: OPAL output for species-level profiling performance across three metagenomic benchmarking datasets.

	ZymoBIOMICS Even GridION (ERR3152364)		ZymoBIOMICS Log GridION (ERR3152366)	
	CPU	GPU	CPU	GPU
metaFlye	3:12		4:05	
Racon x4	5:20	1:08	7:27	1:22
Medaka	1:51	0:16	0:57	0:19
Homopolish	0:46		0:22	
**Total**	11:09	5:22	12:51	6:08

Supplementary Table 4: Cloud-accelerated assembly and polishing times, comparing with and without a GPU. Racon was run with 48 CPUs, and 4 GPUs (Nvidia T4) for the GPU evaluation. Medaka was run with 64 CPUs, and 1 GPU (Nvidia T4) for the GPU evaluation. Threads were set to 64, and batch size was set to 75.

Supplementary Figures

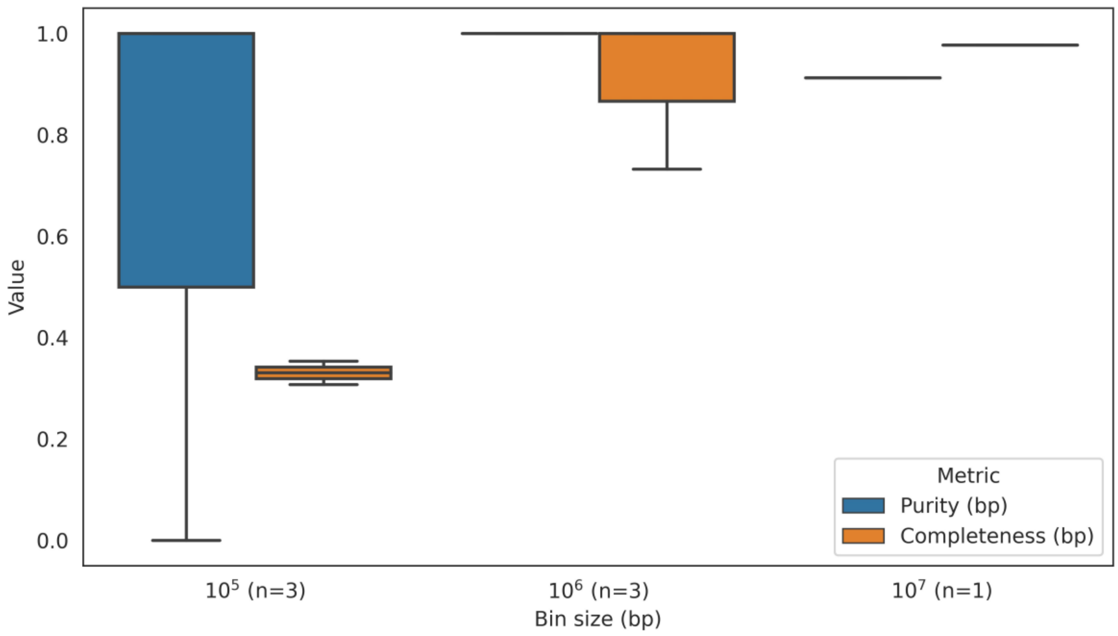

Supplementary Figure 1: Bin purity and completeness for Zymo Even, Log and Gut datasets, stratified by BugSplit-constructed bin size. Whiskers reflect range, rectangles reflect interquartile range, and the solid line reflects median.

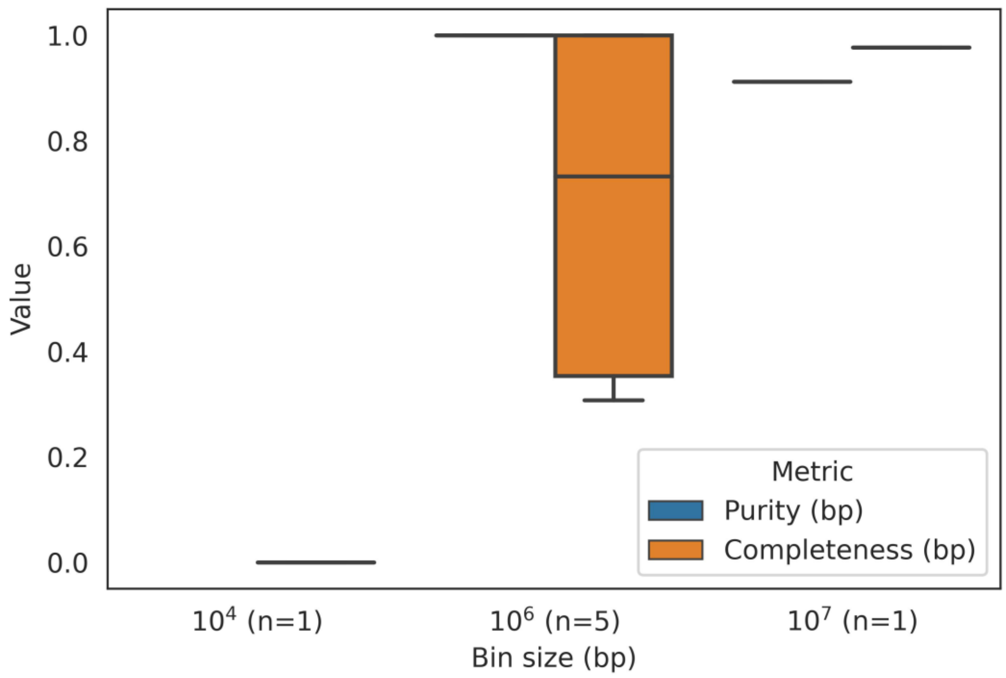

Supplementary Figure 2: Bin purity and completeness for Zymo Even, Log and Gut datasets, stratified by true bin size. Whiskers reflect range, rectangles reflect interquartile range, and the solid line reflects median.

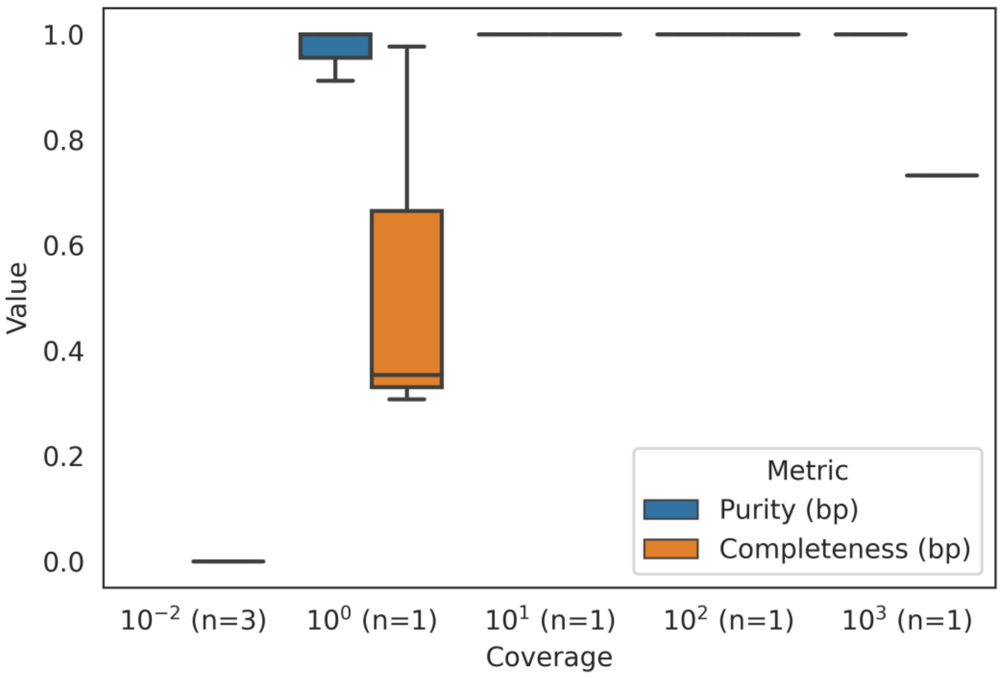

Supplementary Figure 3: Bin purity and completeness for Zymo Even, Log and Gut datasets, stratified by sequencing coverage. Whiskers reflect range, rectangles reflect interquartile range, and the solid line reflects median.

Supplementary Note 1

The following commands were used to execute each program:

qcat: `qcat -f INPUT.fastq --detect-middle -b output --trim --filter-barcodes`

prinseq-lite: `prinseq-lite.pl -fastq INPUT.fastq -out_good OUT.fastq -out_bad null -ns_max_p 10 -min_qual_mean 7 -lc_method dust -lc_threshold 7 -min_len 100`

metaFlye: `flye --nano-raw INPUT.fastq --out-dir fly_output --plasmids --meta --keep-haplotypes --trestle`

Racon: `racon -m 8 -x -6 -g -8 -w 500 --include-unpolished -t THREADS reads.fastq overlaps.paf assemfly.fna`

Medaka: `medaka_consensus -i INPUT.fastq -d ASSEMBLY.fna -o output -m MEDAKA_MODEL`

Homopolish: `homopolish polish -a ASSEMBLY.fna -s mash_sketch.msh -m HOMOPOLISH_MODEL -o OUTPUT_DIR`

Minimap2: `minimap2 -a -t THREADS --split-prefix temp nt.mmi contigs.fa`

DIAMOND (v2.0.9) was run with the commands:

`diamond blastx -q ASSEMBLY.fna -d nr.dmnd -o ASSEMBLY.daa -F 15 -f 100 --range-culling --top 10 -p 96 -b10 -c1`

`daa-meganizer -i ASSEMBLY.daa -mdb megan-nucl-Jan201.db --longReads --threads 96 --minSupportPercent 0`

MMseqs2: `mmseqs taxonomy contig nr assignments tmpFolder --tax-lineage 2 --majority 0.5 --vote-mode 1 --lca-mode 3 --orf-filter 1 --threads 96`

AMBER: `amber.py -p 1 -o zymo_even --ncbi_nodes_file nodes.dmp --ncbi_names_file names.dmp -g gold_standard.bbx *.binning`

OPAL: `opal.py -o OUTPUT -g GOLD_STANDARD.profile *.profile`
