# Supplementary material for: BugSplit enables genome-resolved metagenomics through highly accurate taxonomic binning of metagenomic assemblies
Source: Commun Biol. 2022 Feb 22;5:151. doi: 10.1038/s42003-022-03114-4 (PMC8864044; doi:10.1038/s42003-022-03114-4)
Supplement: Supplementary file 2 — Description of Additional Supplementary Files [file 42003_2022_3114_MOESM2_ESM.pdf]

Description of Additional Supplementary Files

File name: Supplementary Data 1
Description: Figure 1a-b raw data.

File name: Supplementary Data 2
Description: Figure 1c raw data.

File name: Supplementary Data 3
Description: Figure 2a-c raw data.
