# Supplementary material for: BugSplit enables genome-resolved metagenomics through highly accurate taxonomic binning of metagenomic assemblies
Source: Commun Biol. 2022 Feb 22;5:151. doi: 10.1038/s42003-022-03114-4 (PMC8864044; doi:10.1038/s42003-022-03114-4)
Supplement: Supplementary file 6 — Reporting Summary [file 42003_2022_3114_MOESM6_ESM.pdf]

nature portfolio

Corresponding author(s): Samuel Chorlton

Last updated by author(s): Jan 6, 2022

Reporting Summary

Nature Portfolio wishes to improve the reproducibility of the work that we publish. This form provides structure for consistency and transparency in reporting. For further information on Nature Portfolio policies, see our Editorial Policies and the Editorial Policy Checklist.

Statistics

For all statistical analyses, confirm that the following items are present in the figure legend, table legend, main text, or Methods section.

n/a	Confirmed	
☐	☒	The exact sample size (n) for each experimental group/condition, given as a discrete number and unit of measurement
☐	☒	A statement on whether measurements were taken from distinct samples or whether the same sample was measured repeatedly
☐	☒	The statistical test(s) used AND whether they are one- or two-sided<br>*Only common tests should be described solely by name; describe more complex techniques in the Methods section.*
☐	☒	A description of all covariates tested
☐	☒	A description of any assumptions or corrections, such as tests of normality and adjustment for multiple comparisons
☐	☒	A full description of the statistical parameters including central tendency (e.g. means) or other basic estimates (e.g. regression coefficient) AND variation (e.g. standard deviation) or associated estimates of uncertainty (e.g. confidence intervals)
☐	☒	For null hypothesis testing, the test statistic (e.g. F, t, r) with confidence intervals, effect sizes, degrees of freedom and P value noted<br>*Give P values as exact values whenever suitable.*
☐	☒	For Bayesian analysis, information on the choice of priors and Markov chain Monte Carlo settings
☐	☒	For hierarchical and complex designs, identification of the appropriate level for tests and full reporting of outcomes
☐	☒	Estimates of effect sizes (e.g. Cohen's d, Pearson's r), indicating how they were calculated

Our web collection on statistics for biologists contains articles on many of the points above.

Software and code

Policy information about availability of computer code

Data collection	Data was downloaded from NCBI Sequence Read Archive with the NCBI SRA Toolkit version 2.11.0.
Data analysis	The commands underlying the BugSplit algorithm are detailed in the supplementary material, and a free, hosted version of BugSplit (version 2.1) is available at https://bugseq.com/academic. Additionally, executable code has been archived at Zenodo: https://doi.org/10.5281/zenodo.5826348. The modified ResFinder/PointFinder code is available at https://bitbucket.org/genomicepidemiology/resfinder (version 4.2). OPAL (version 1.0.10) and AMBER (version 2.0.2) were downloaded from their respective GitHub pages (https://github.com/CAMI-challenge/OPAL and https://github.com/CAMI-challenge/AMBER).

For manuscripts utilizing custom algorithms or software that are central to the research but not yet described in published literature, software must be made available to editors and reviewers. We strongly encourage code deposition in a community repository (e.g. GitHub). See the Nature Portfolio guidelines for submitting code & software for further information.

Data

Policy information about availability of data

All manuscripts must include a data availability statement. This statement should provide the following information, where applicable:

- Accession codes, unique identifiers, or web links for publicly available datasets
- A description of any restrictions on data availability
- For clinical datasets or third party data, please ensure that the statement adheres to our policy

Benchmarking data was downloaded from:
Bacillus anthracis whole genome nanopore sequencing: SRA accession SRR10088696
ZymoBIOMICS Even nanopore mNGS: SRA accession ERR3152364

ZymoBIOMICS Log nanopore mNGS: SRA accession ERR3152366
ZymoBIOMICS Gut PacBio HiFi mNGS: SRA accession SRR13128014
CAMI High Complexity gold standard assembly and ground truth labels: https://openstack.cebitec.uni-bielefeld.de:8080/swift/v1/CAMI_I_HIGH using the CAMI downloader.
Hypervirulent Klebsiella pneumoniae nanopore mNGS data: NCBI BioProject PRJNA663005
Neisseria gonorrhoeae nanopore mNGS data: NCBI BioProject PRJEB35173
NCBI nt database from 2019: https://openstack.cebitec.uni-bielefeld.de:8080/swift/v1/CAMI_2_DATABASES/ncbi_blast/nt.gz

Newly generated COVID-19 nanopore mNGS data has been deposited under NCBI Bioproject Accession Number PRJNA766077.

Field-specific reporting

Please select the one below that is the best fit for your research. If you are not sure, read the appropriate sections before making your selection.

☒ Life sciences ☐ Behavioural & social sciences ☐ Ecological, evolutionary & environmental sciences

For a reference copy of the document with all sections, see nature.com/documents/nr-reporting-summary-flat.pdf

Life sciences study design

All studies must disclose on these points even when the disclosure is negative.

Sample size	No sample size calculation was performed. All data publicly available for each validation test case was used. A convenience set of 3 samples was used to simulate the novel pandemic pathogen application with SARS-CoV-2 mNGS sequencing.
Data exclusions	No data was excluded from analysis.
Replication	Methods were applied to diverse sample types and pathogens to demonstrate replicability. Samples were not analyzed twice given identical results with the bioinformatic analyses.
Randomization	Not applicable as this was not an interventional study.
Blinding	Investigators were not blinded to the origin of validation test data.

Reporting for specific materials, systems and methods

We require information from authors about some types of materials, experimental systems and methods used in many studies. Here, indicate whether each material, system or method listed is relevant to your study. If you are not sure if a list item applies to your research, read the appropriate section before selecting a response.

Materials & experimental systems

n/a	Involved in the study	
☒	☐	Antibodies
☒	☐	Eukaryotic cell lines
☒	☐	Palaeontology and archaeology
☒	☐	Animals and other organisms
☐	☒	Human research participants
☒	☐	Clinical data
☒	☐	Dual use research of concern

Methods

n/a	Involved in the study	
☒	☐	ChIP-seq
☒	☐	Flow cytometry
☒	☐	MRI-based neuroimaging

Human research participants

Policy information about studies involving human research participants

Population characteristics	No clinical information on human participants was available to study researchers. Samples were anonymized before inclusion in this study.
Recruitment	A convenience sample of two nasopharyngeal swabs with remaining material, after primary clinical testing, was used for this study. Full details of the samples, selection and ethics approval is available at https://doi.org/10.1101/2021.08.13.21261922
Ethics oversight	University of British Columbia Research Ethics Board (H20-02152)

Note that full information on the approval of the study protocol must also be provided in the manuscript.
